# Real-World Experience of Using Etelcalcetide for Secondary Hyperparathyroidism in Community-Based Hemodialysis Centers in Singapore

**DOI:** 10.7759/cureus.48186

**Published:** 2023-11-02

**Authors:** Behram A Khan, XiaoJie Qu, Yan Hua, Muhammad M Javaid

**Affiliations:** 1 Medicine, National University of Singapore, Singapore, SGP; 2 Medical Affairs, The National Kidney Foundation Singapore, Singapore, SGP; 3 Medicine, Monash University, Melbourne, AUS; 4 Medicine, Deakin University, Warrnambool, AUS; 5 Nephrology, Woodlands Health, Singapore, SGP

**Keywords:** calcium phosphate product, mineral bone disease, chronic kidney disease-mineral and bone disorder, parathyroid hormone (pth), intact parathyroid hormone, calcimimetic, etelcalcetide, chronic hemodialysis, hemodialysis, secondary hyperparathyroidism

## Abstract

Introduction

Chronic kidney disease-related mineral and bone disorder (CKD-MBD), characterized by abnormalities in calcium, phosphate, and parathyroid hormone metabolism, with impaired bone turnover and extravascular calcification is a known complication of advanced chronic kidney disease (CKD). Secondary hyperparathyroidism (SHPT) develops early in the disease and its prevalence gradually increases with the disease progression, becoming almost universal in patients with end-stage renal disease (ESRD). The treatment for SHPT includes synthetic vitamin D analogs, calcitriol or calcimimetics. Recently, intravenous etelcalcetide was introduced as a second-generation calcimimetic. This article provides the real-world experience of using etelcalcetide in multiethnic Asian patients receiving hemodialysis at community-based hemodialysis centers in Singapore.

Methods

This study was real-world evidence, generated by a retrospective clinical audit of routine clinical care of hemodialysis patients in community-based centers in Singapore who received etelcalcetide for treating SHPT. The information on the starting and maximum dose of etelcalcetide, duration of treatment on hemodialysis, parathyroid hormone (PTH) levels, dialysate calcium, concomitant medications, and reasons for discontinuation were collected from the medical records. PTH levels were collected at four-, eight-, and twelve-month intervals.

Results

A total of 148 patients received etelcalcetide during the study period. Ten patients died and twenty discontinued their treatment, with 118 patients remaining on treatment. Demographically, the patients included Chinese, Malay, Indians, and those belonging to other racial groups. The starting dose of etelcalcetide ranged from 2.5 mg once per week to 7.5 mg three times a week. There was a 16.8% reduction (p=<0.001) in intact-PTH after four months of therapy. Target intact-PTH level of less than 60 pmol/L, was reported as 1.4% at baseline, with 22.3% at four months (p<0.001) and 25.9% at eight months (p=0.028). Calcium and phosphate levels were also tracked as part of the safety and efficacy measures of using etelcalcetide. No symptomatic hypocalcemia was noted and phosphate levels were noted to decline significantly. Overall, the calcium-phosphate product reduced at four months (13.2%, p=<0.001) and eight months (12.7%, p<0.05). An analysis of concomitant medication usage, dialysate calcium utilized, and the side effects of etelcalcetide were also recorded. Finally, a brief descriptive analysis of the patient's subjective feedback regarding etelcalcetide was also reported, especially regarding the reduction in pill burden and overall compliance to medications.

Conclusion

Etelcalcetide is safe and effective for treating SHPT in multi-ethnic Asian hemodialysis patients and can be considered an alternative to oral cinacalcet. Our study showed no side effects, which was one of the key reasons for non-compliance to traditional calcimimetics. A favorable compliance profile with reduced pill burden was noted by using this intravenous calcimimetic.

## Introduction

End-stage renal disease (ESRD) is a significant healthcare problem worldwide. In Singapore, the incidence of patients requiring dialysis has gradually increased over the years. There were 8668 prevalent dialysis patients in 2021 compared to 7007 five years ago [1]. Eighty-five percent were on hemodialysis, with over 95% receiving treatment at community-based centers [1].

Chronic kidney disease-related mineral and bone disorder (CKD-MBD), characterized by abnormalities in calcium, phosphate, and parathyroid hormone metabolism, with impaired bone turnover and extravascular calcification is a known complication of advanced chronic kidney disease (CKD) [2]. Secondary hyperparathyroidism (SHPT) develops early in the disease and its prevalence gradually increases with the disease progression, becoming almost universal in patients with end-stage renal disease (ESRD) [2, 3]. Poorly controlled SHPT increases fracture risk, cardiovascular disease, and mortality [4]. The treatment of hyperphosphatemia, maintaining normal calcium levels, and keeping serum parathyroid (PTH) levels within two to nine times the normal upper limit by synthetic vitamin D analogs, calcitriol or calcimimetics are the recommended management strategies for SHPT [2].

Oral cinacalcet, a first-generation calcimimetic, has been clinically used for several years. More recently, intravenous etelcalcetide was introduced as a second-generation calcimimetic, with its safety and efficacy being reported in three randomized controlled trials [5,6]. However, those trials primarily included Caucasian and Black patients, with the effectiveness of etelcalcetide in the Asian population remaining largely unstudied. This article provides the real-world experience of using etelcalcetide in multiethnic Asian patients receiving hemodialysis at community-based hemodialysis centers in Singapore.

## Materials and methods

Study design and subjects

This study was real-world evidence, generated by a retrospective clinical audit of routine clinical care of hemodialysis patients in community-based centers in Singapore who received etelcalcetide for treating SHPT. This study design is exempt from institutional review board approvals and complies with the necessary data handling policies of the centers involved. Specifically, the investigators used aggregated data sets with de-identified patient data for this study. Patients who received etelcalcetide as part of their CKD-MBD and SHPT management between April 2021 and July 2022 were included in the study.

Data collection

Data collection was done retrospectively as a one-time exercise according to the criteria and parameters defined by the audit team. All patients receiving etelcalcetide during the study period were included in the audit. Patients’ demographic information and health-related variables were obtained from a clinical dashboard, which extracted aggregated and de-identified data from the electronic medical records. The information on the starting and maximum dose of etelcalcetide, duration of treatment on hemodialysis, PTH levels, dialysate calcium, concomitant medications, and reasons for discontinuation were collected from the medical records. PTH levels were collected were collected at four-, eight-, and twelve-month intervals.

Statistical analysis

The results were analyzed by computing mean values with standard deviation computations and median values with inter-quartile range (IQR). The p-values were calculated using paired Student’s t-test, Wilcoxon signed rank test, exact McNemar’s test, and Fisher’s exact test. These tests were applied based on the data set being analyzed and indicated clearly in the figures where the statistical analysis was being performed.

All patients who experienced any adverse events after etelcalcetide initiation or were deceased during the audit period were reviewed to provide a descriptive account of the findings. Statistical significance was defined as p<0.05 and was highlighted with an asterisk in the figures and shown in bold for better visualization.All statistical analysis was performed using IBM SPSS statistics for Windows version 26.0 software (IBM Corp., Armonk, USA).

## Results

Characteristics of participants

**Figure 1:**
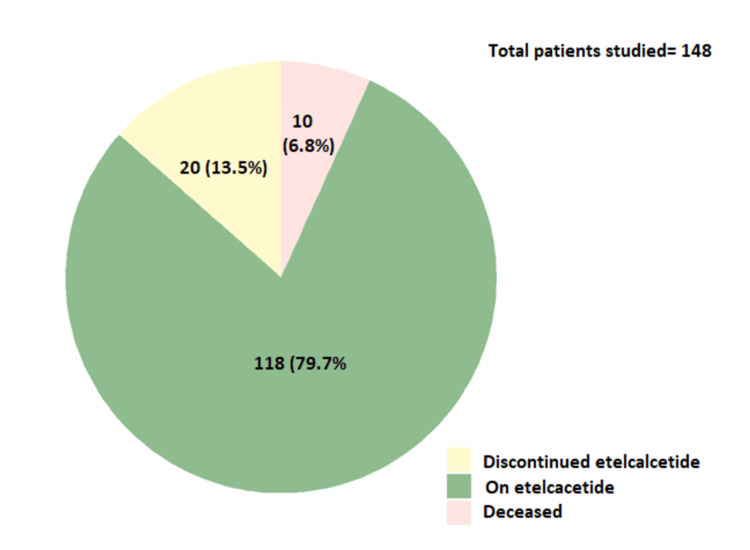
Number of patients receiving or having received etelcalcetide Time frame: April 2021 to July 2022

A total of 148 patients received etelcalcetide during the study period. Ten patients died and 20 discontinued their treatment. At the end of the study period, 118 patients remained on treatment (Figure 1).

Demographically 58.1% were Chinese, 34.5% Malay, 6.1% Indians, and 1.4% belonged to the other racial groups. The median age was 61.5 years (Interquartile range, IQR: 52.3, 69.5). The gender distribution comprised 57.4% males and 42.6% females.

A total of 54 patients (36.5%) received treatment for less than four months, 66 patients (44.6%) between 4 and 8 months, 27 patients (18.2%) between 8 and 12 months, and only one patient (0.7%) received etelcalcetide for more than 12 months. The mean duration of treatment was 5.28 ± 3.06 months and the median was 5.70 (IQR: 2.00, 7.30).

Outcomes

**Figure 2:**
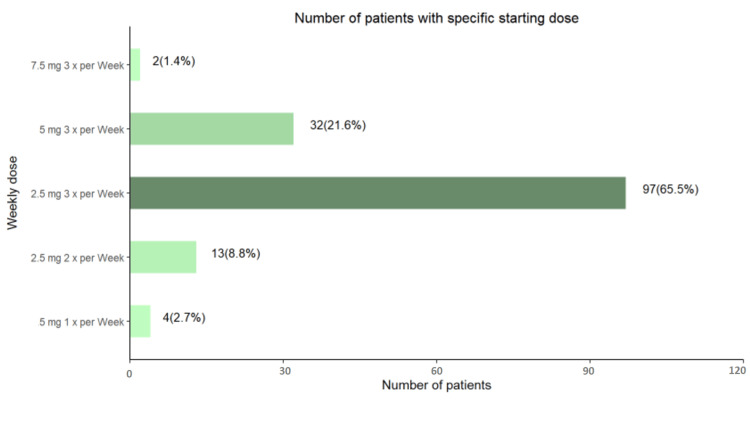
Number of patients with specific starting dosages of etelcalcetide mg: milligram; Mean ± SD: 8.97 ± 3.79; Median (IQR): 7.5 (7.5, 7.5)

The starting dose ranged from 2.5 mg once per week to 7.5 mg three times a week (Mean ± SD: 8.97 ± 3.79 and Median 7.5, IQR: 7.5, 7.5). A majority of 97 patients (65.5%) patients had a starting dose of 2.5 mg three times a week (Figure 2).

**Figure 3:**
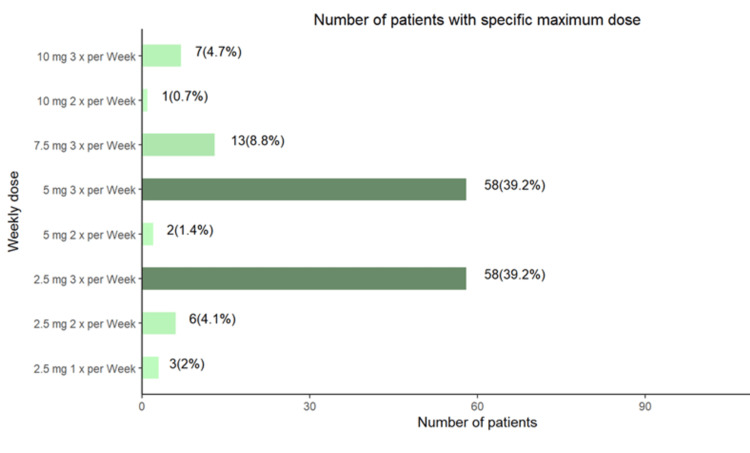
Number of patients with specific maximum dosages of etelcacetide mg: milligram; Mean ± SD: 12.74 ± 6.36; Median (IQR): 15 (7.5, 15)

The maximum weekly dose ranged from 2.5 mg once a week to 10 mg three times a week (Mean ± SD: 12.74 ± 6.36. Median 15 (IQR: 7.5, 15) (Figure 3).

The mean intact-PTH at baseline was 147 ± 72.9 pmol/L and the median was 128 pmol/L (IQR: 107, 172). At four months, the mean intact-PTH reduced to 118 ± 84.4 pmol/L. There was a 16.8% reduction (p=<0.001) in intact-PTH after four months of therapy (Table 1).

**Table 1:** Parathyroid hormone level in relation to etelcalcetide usage duration iPTH: intact parathyroid hormone; pmol/L: picomoles per liter ** p<0.001; ^#^ One patient was on etelcalcetide for 8 months but the latest parathyroid hormone level result was not available at the end of the study period. P-values were calculated at month 4 by using paired Student's t-test and that of month 8 by using Wilcox signed rank test.

	Initiation	Month-4	Month-8	Month-12
Number of patients	148	94	27^#^	1
Mean iPTH (pmol/L) Mean ± SD	147 ± 72.9	118 ± 84.4	145 ± 107	104
Median iPTH(pmoI/L) Median (IQR)	128 (107, 172)	97.7 (62.0, 148)	113 (68.4, 214)	104
iPTH reduction (absolute, pmoI/L)	Reference	28.3**	23.4	353
iPTH reduction (percentage, %)	Reference	16.8**	15.0	77.2

Furthermore, when assessing for the institutional target intact-PTH level of less than 60 pmol/L, we see a progressive decline in levels into the target range from 1.4% at baseline to 22.3% at four months and 25.9% at eight months. When compared with patients who were out of the target range, this reduction was statistically significant with a p-value of <0.001 at four months and a p-value of 0.028 at eight months. Table 2 below further illustrates these points.

**Table 2:** Parathyroid hormone level ranges, in relation to etelcalcetide usage duration iPTH: intact parathyroid hormone; pmol/L: picomoles per liter ^#^ One patient was on etelcalcetide for 8 months, but the latest PTH result was unavailable at the end of the study; * p<0.05, calculated by using exact McNemar's test; ** p<0.001, calculated by using paired Student's t-test.

	Initiation	Month-4	Month-8	Month-12
Number of patients	148	94	27^#^	1
Number of patients with iPTH <60 pmol/L	2 (1.4%)	21(22.3%)	7 (25.9%)	0
Number of patients with iPTH 60-100 pmol/L	31 (20.9%)	27 (28.7%)	3 (11.1%)	0
Number of patients with iPTH >100 pmol/L	115 (77.7%)	46 (48.9%)	17 (63.0%)	1 (100.0%)
p-value	Reference	<0.001**	0.028*	NA

We also specifically looked at patients with no reduction in PTH and the possible reasons behind such findings. At four and eight months, 32 (34%) and 10 (37%) of patients, respectively, had no reduction in their intact-PTH levels. We looked at various demographic factors, including age, gender, and race to further study if any factors contributed to this finding. Table 3 below illustrates this data, and the only statistically significant finding of non-responders was Malay ethnicity at the 8-month timeframe.

**Table 3:** Summary of patients with and without reduction in intact parathyroid hormone levels at 4-month and 8-month timeframes. * p<0.05 P-values were calculated by comparing patients with and without reduction at each timeframe. Continuous variables were summarized as median (interquartile range) and compared using the Wilcox rank sum test. Categorical variables were summarized as count (percentage) and compared using Fisher's exact test.

Variables	All patients (n = 148)	With reduction at month-4 (n=62)	Without reduction at month-4 (n=32)	With reduction at month-8 (n=17)	Without reduction at month-8 (n=10)
Age at medication initiation (year)	61.5 (52.3, 69.5)	60.2 (49.3, 68.9)	62.9 (53.6, 69.4)	62.6 (51.0, 69.0)	51.9 (43.8, 56.2)
Gender (Female)	63 (42.6%)	26 (41.9%)	14 (43.8%)	9 (52.9%)	4 (40%)
Race*					
Chinese	86 (58.1%)	37 (59.7%)	15 (46.9%)	9 (52.9%)	1(10%)
Malay	51 (34.5%)	18 (29.0%)	16 (50%)	5 (29.4%)*	8 (80%)*
Indian	9 (6.1%)	6 (9.7%)	1(3.1%)	2 (11.8%)	1(10%)
Others	2 (1.4%)	1(1.6%)	0 (0%)	1(5.9%)	0 (0%)

As different dosages of etelcalcetide were used for various patients during this retrospective clinical audit, an assessment of the results was also made to compare the dosages that did result in the reduction of PTH, in comparison to the dosages that did not. As serum phosphate level would also affect effect the PTH level, as well as act as a surrogate marker for compliance in general, this was added to the results for analysis. Table 4 summarizes these findings, with the statistically significant ones shown as bold numbers.

**Table 4:** Summary of patients with and without reduction in intact parathyroid hormone and phosphate levels at 4-month and 8-month timeframes. Summary of dosages of etelcalcetide usage indicated. * p<0.05 iPTH: intact parathyroid hormone; pmol/L: picomoles per liter; mg: milligram; PO4: phosphate P-values were calculated by comparing patients with and without reduction at each timeframe. Continuous variables were summarized as median (interquartile range) and compared using Wilcox rank sum test. Categorical variables were summarized as count (percentage) and compared using Fisher's exact test.

Variables	All patients (n = 148)	With reduction at month-4 (n=62)	Without reduction at month-4 (n=32)	With reduction at month-8 (n=17)	Without reduction at month-8 (n=10)
Baseline iPTH (pmol/l)	128 (107, 172)	119 (97.5, 177)	127 (96.5, 168)	128 (106, 191)	171 (94.7, 22.0)
Start dose (mg/week)	7.50 (7.50, 7.50)	7.50 (7.50, 7.50)	7.50 (7.50, 15.0)	7.50 (7.50, 150)	7.50 (7.50, 13.1)
Month-4 dose (mg/week)	15.0 (7.50, 15.0)	12 . 5 (7.50, 15.0)*	15.0 (7.50, 16.9)*	15.0 (7.50, 15.0)	15.0 (7.50, 15.0)
Month-8dose (mg/week)	15.0 (9.38, 15.0)	NA	NA	15.0 (7.50, 15.0)	15.0 (15.0, 20.6)
Maximum dose (mg/week)	15.0 (7.50, 15.0)	15.0 (7.50, 15.0)*	15.0 (15.0, 22.5)*	15.0 (7.50, 15.0)	17.5 (15.0, 22.5)
Baseline P04 ( mg/dL)	5.20 (4.30, 6.10)	5.20 (4.43, 5.80)	5.55 (4.55, 6.20)	5.30 (4.70, 5.80)8*	6.25 (6.10, 6.30)*
Month-4 P04 (mg/dl)	4.30 (3.50, 5.30)	3.90 (3.20, 4.80)*	5.00 (4.18, 5.85)*	4.50 (3.20, 5.10)*	5.85 (4.75, 6.80)*
Month-8P04 (mg/dl)	5.10 (4.38, 5.73)	NA	NA	4.60 (3.80, 5.20)	5.50 (5.03, 5.98)

Calcium levels were measured and analyzed. At baseline, the mean serum calcium measured 9.50 ± 0.573 mg/dL, which remained relatively stable from a safety assessment aspect during the study period. 12.8% of patients at month four and 21.4% at month eight developed hypocalcemia with serum Ca levels below 8.4 mg/dL. No symptoms or signs were reported in any of the patients with such levels and no cardiac or other complications were identified. Table 5 illustrates the change in calcium levels.

**Table 5:** Summary of calcium levels and their reduction, in reference to usage duration of etelcalcetide. The data of serum calcium (Ca) levels in milligrams per deciliter (mg/dL) has been represented as mean with standard deviation (SD) and median with interquartile range (IQR). The data for the reduction in serum calcium levels has been represented as absolute reduction and percentage (%) reduction. ** p<0.001

	Initiation	Month-4	Month-8	Month-12
Number of patients	148	94	28	1
Mean Ca (mg/dL) Mean ± SD	9.50 ± 0.573	8.96 ± 0.569	9.00 ± 0.696	9.10
Median Ca (mg/dL) Median (IQR)	9.60 (9.00, 9.90}	8.90 (8.60, 9.38}	9.00 (8.65, 9.22)	9.10
Ca reduction (absolute, mg/dL)	Reference	0.514**	0.300	0.700
Ca reduction (percentage, %)	Reference	5.19**	2.99	7.14
Number of patients with Ca < 8.4mg/dL	2 (1.4%)	12 (12.8%)	6 (21.4%)	0

Both phosphate and calcium-phosphate product (Ca-PO4) was studied. Absolute phosphate levels declined significantly, whereas there was a significant reduction in Ca-Po4 product reduction at four months (13.2%, p=<0.001) and eight months (12.7%, p<0.05). Table 6 and Table 7 below illustrate these findings, respectively.

**Table 6:** Phosphate levels and their reduction, in reference to the duration of usage of etelcalcetide. The data of serum phosphate (PO4) levels in milligrams per deciliter (mg/dL) has been represented as mean with standard deviation (SD) and median with interquartile range (IQR). The data for the reduction in serum phosphate levels has been represented as absolute reduction and percentage (%) reduction. * p<0.05; ** p<0.001

	Initiation	Month-4	Month-8	Month-12
Number of patients	148	94	28	1
Mean PO4 (mg/dL) Mean ± SD	5.21 ± 1.38	4.52 ±1.36	4.91 ±1.06	4.90
Median PO4 (mg/dL) Median (IQR)	5.20 (4.30, 6.10)	4.30 (3.50, 5.30)	5.10 (4.38, 5.73)	4.90
PO4 reduction (absolute, mg/dL)	Reference	0.684**	0.750*	0.200
PO4 reduction (percentage, %)	Reference	8.32**	11.4	3.92
Number of patients with PO4 > 5.5 mg/dL	60 (40.5%)	21 (22.3%)	8 (28.6%)	0

**Table 7:** Calcium phosphate product levels and their reduction, in reference to the duration of usage of etelcalcetide. The data of calcium phosphate product (Ca-PO4) in milligrams per deciliter squared (mg^2^/dL^2^) has been represented as mean with standard deviation (SD) and median with interquartile range (IQR). The data for the reduction in calcium phosphate product has been represented as absolute reduction and percentage (%) reduction. * p<0.05; ** p<0.001

	Initiation	Month-4	Month-8	Month-12
Number of patients	148	94	28	1
Ca-PO4 product (mg^2^/dL^2^) Mean ± SD	49.5 ±13.0	40.4 ±12.1	44.1 ±9.88	44.6
Ca-PO4 product (mg^2^/dL^2^) Median (IQR)	49.7 (41.7, 58.1)	37.9 (31.7, 47.4)	46.5 (38.5, 52.0)	44.6
Ca-PO4 reduction (absolute, mg^2^/dL^2^)	Reference	8.83**	8.19*	5.39
Ca-PO4 reduction (percentage, %)	Reference	13.2**	12.7*	10.8
Number of patients with Ca-PO4 > 55 mg^2^/dL^2^	49 (33.1%)	12 (12.8%)	3 (10.7%)	0

Regarding concomitant medication, eight (5.4%) patients were only on a single calcium-based phosphate binder, and seven (4.7%) were on a single non-calcium-based phosphate binder. There were six (4.1%) patients were on alfacalcidol only. Also, six (10.8%) patients were on a combination of a non-calcium-based phosphate binder and a calcium-based phosphate binder; 24 (16.2%) on a combination of alfacalcidol and a calcium-based phosphate binder, and 35 (23.6%) on alfacalcidol and a non-calcium-based phosphate binder. Furthermore, 52 (35.1%) patients received three types of concomitant medication.

Dialysate calcium as a variable was studied and it was found that on average 62.5% of patients were on low calcium dialysate (1.25 mmol/l) and 38.5% on normal calcium dialysate (1.5 mmol/l). 

Of the twenty patients who discontinued the etelcalcetide treatment, eight had side effects. This included body weakness, inability to tolerate medication, shortness of breath, headache, and bone pain in four patients. Also, over-suppressed PTH level was the reason in two patients, as well as hypocalcemia and possible allergic reaction in one patient each. Parathyroid surgeries were performed on four patients which resulted in the cessation of etelcalcetide therapy and two patients stopped treatment due to cost. One patient who developed hypocalcemia was on etelcalcetide for eight days at a dose of 5 mg three times a week. Calcium before medication initiation was already low at 8.3mg/dL. Calcium measured eight days after initiation was 7.9 mg/dL. Medication was discontinued by the attending nephrologist. The patient did not have any signs or symptoms of hypocalcemia and was not hospitalized. The calcium after discontinuation improved to 8.5 mg/dL. No cause was documented in the clinical notes in the rest of the five patients who were discontinued from this medication.

A descriptive analysis of the case notes during the audit did reveal a general appreciation by the patients in switching from an oral to intravenous medication, which on a subjective level increased their compliance to their overall treatment regimen, with polypharmacy being one of the key complaints of most patients. Due to the descriptive nature of such a review, it was not feasible to numerically report or analyze the subjective feedback.

This retrospective clinical audit was done during the end of the SARS-CoV-2 pandemic in Singapore. Telemedicine was not being practiced at these centers at that time and routine rounding of attending nephrologists had commenced in the dialysis centers being studied [7,8]. No adverse outcomes in general have been noted regarding the overall hemodialysis care of these patients in relation to any infection control measures related to the SARS-CoV-2 pandemic [9,10].

## Discussion

Calcium-sensing receptors (CaSR) play a central role in regulating PTH. Ionized calcium (Ca) is the primary agonist of the CaSR. Ca activates the receptors, causing inhibition of PTH production and release, thereby preventing the parathyroid gland’s hypertrophy. However, CaSR is a low-selectivity structure and apart from Ca, it can also be activated by various other divalent or trivalent cations or other compounds such as neomycin, spermine, and amino acids [11]. Several compounds termed calcimimetics, capable of modulating the CaSR, have been identified. Type I calcimimetics mimics the effect of extracellular Ca in the CaSR, while type II calcimimetics causes structural alternations in CaSR, thus increasing their sensitivity to extracellular Ca [11].

Cinacalcet, the first-generation oral calcimimetic agent, has been in clinical use for SHPT in ESRD patients on dialysis for several years [2]. Cinacalcet decreases the PTH secretion by increasing the sensitivity of CaSR to the extracellular level in a dose-dependent manner and as a result, also reduces the serum phosphate and Ca levels [12]. The EVOLVE trial examined the impact of cinacalcet usage in hemodialysis patients. Cinacalcet was associated with a decreased risk of cardiovascular death and fracture risk in patients over 65 of age. However, these benefits were not observed in the younger patients [13, 14]. Hypocalcemia and gastrointestinal symptoms were the main side effects, which were the main reasons for the high dropout rate in the cinacalcet group in the 2012 EVOLVE trial, potentially resulting in a negative study [15].

Etelcalcetide is a second-generation calcimimetic that is given intravenously and reduces the PTH level by binding to the CaSR and enhancing the receptor activation by extracellular calcium. The agent was primarily developed to increase compliance by reducing gastrointestinal side effects [4]. The efficacy of etelcalcetide was assessed in two parallel, phase 3, randomized, placebo-controlled trials involving 1023 hemodialysis patients with its effect on moderating SHPT. The mean age was 58.2 ± 14.4 years, and 60.4% were men. The primary endpoint was the proportion of patients with greater than 30% reduction from baseline in mean PTH during weeks 20-27. Compared to placebo, etelcalcetide resulted in a more significant decrease in serum PTH over 26 weeks. In trial A, 188 (74.0%) patients in the treatment arm compared to 21 (8.3%) in the placebo arm, with a p-value of <0.001, showed this decrease in serum PTH. In trial B, there were 192 (75.3%) patients in the treatment arm compared to 25 (9.6%) patients in the placebo arm, with a p-value of <0.001 for this reduction in PTH levels. Furthermore, 49.6% of patients compared to 5.1% in trial A (p<0.001) and 53.3% compared to 4.6% of patients in trial B (p<0.001) achieved the secondary endpoint proportion of patients achieving mean PTH levels ≤30 pg/L. Hypocalcemia, defined as a serum Ca level less than 8.3 mg/dL, was seen in 61% of patients in trial A and 66.7% in trial B. Median dose of etelcalcetide was 7.1 mg per dialysis session (IQR, 3.6-10.0 mg) in trial A and 5.0 mg (IQR, 2.5-10.0 mg) in trial B [5]. Block et al. compared the effectiveness of etelcalcetide versus cinacalcet and concluded that the use of etelcalcetide in hemodialysis patients with moderate to severe SHPT was not inferior to cinacalcet [6].

Our real-world experience by performing a clinical audit showed that etelcalcetide was safe and effective in treating SHPT in multi-ethnic Asian hemodialysis patients. Nearly 65% of patients showed a reduction in PTH level compared to the baseline. More than 85% of patients could continue their treatment and only 20 dropped out for various reasons. The incidence of side effects was low in our cohort of patients; more importantly, none reported significant gastrointestinal side effects. This contrasted with the previously reported studies, where some gastrointestinal side effects were identified. In the two randomized trials reported by Block et al., up to 14% of patients had diarrhea, 12% nausea, and 10% had vomiting. Hypocalcemia (serum Ca <8.4 mg/dL) was also significantly lower in our patients as compared to the reported randomized trials [5].

In our group of patients, a sub-analysis showed 42.5% who completed four months of treatment and 37% of patients who received treatment for eight months achieved more than a 30% reduction in PTH levels. This was significantly lower than 75% of patients achieving the primary endpoint of more than 30% reduction in PTH at 26 weeks reported in the previous trials [5]. However, it may not be feasible to make a direct comparison, as our study reports the real-world experience of patients receiving etelcalcetide in community hemodialysis centers, which differs from the controlled trial environment. Furthermore, the number of patients completing more than eight months of treatment in our cohort was significantly lower (19%), which might have skewed the results. Moreover, our patients' median weekly etelcalcetide dose was considerably lower (10.5 mg versus 21 mg on trial A and 15 mg on trial B), which might be another contributing factor. The reason for this may be that in a real-world setting, nephrologists may be more cautious in utilizing a new medication, in comparison to a controlled trial where the dosages are determined largely by the study protocols.

Of the 94 patients who completed four months of treatment, 32 had no reduction in their PTH levels. Subsequently, 10 patients were non-responders among 27 patients at the eight-month mark. Due to our clinical audit's retrospective nature, we could not identify a definitive cause. Some confounders that may have contributed to this finding include attending nephrologists’ inexperience with using etelcalcetide, which may have impacted optimal utilization in a real-world setting. Additionally, there may have been some logistical challenges of medication procurement, as it was a non-subsidized drug during this clinical audit. Further studies will be needed to ascertain the causes of some patients' lack of response to etelcalcetide, in a prospective trial setting.

Etelcalcetide was found to be helpful in general to the patient’s overall personal perception of their compliance to their treatment regimens. Polypharmacy and pill-burden is a known issue that hinders compliance to medication and leads to poor clinical outcomes as well as poor quality of life [16]. Our study may demonstrate an overall strategy to use drug alternatives which are available in intravenous forms for chronic hemodialysis patients, to help reduce polypharmacy and pill burden. Examples of these are intravenous iron preparations, activated vitamin D analogues in intravenous forms, and now a calcimimetic that is available in parenteral preparation [17]. Overall, such a broader strategy may significantly improve clinical outcomes for patients suffering from poor clinical outcomes due to non-compliance [18]. Further studies would be needed to study this interesting aspect of the overall clinical management of chronic hemodialysis patients in the community.

Strengths and limitations

This study’s main strength is the real-world evidence and reporting of using etelcalcetide in multi-ethnic Asian hemodialysis patients in community dialysis centers. Such experience can provide helpful, practical information and insights to nephrologists managing hemodialysis patients in the community. The main limitation is the retrospective design of the audit project without a control group, which makes comparison with other treatments for SHPT in our cohort impractical. Additionally, etelcalcetide was provided via an institution’s internally funded program, with inherent limitations on the quantities available for optimal prescription and usage. Lastly, due to the retrospective audit of patient records, some data points may be missing, which we cannot remedy.

## Conclusions

Etelcalcetide is safe and effective for treating SHPT in multi-ethnic Asian hemodialysis patients and can be considered an alternative to oral cinacalcet, where compliance with daily oral dose is a problem. Our study showed no gastrointestinal side effects, which was one of the key reasons for non-compliance to traditional calcimimetic, in addition to improving compliance by reducing the pill burden and polypharmacy, which is rampant in end-stage renal disease patients' treatment regimens.
